# Proto-oncogene c-Myb potentiates cisplatin resistance of ovarian cancer cells by downregulating lncRNA NKILA and modulating cancer stemness and LIN28A-let7 axis

**DOI:** 10.1186/s13048-024-01429-w

**Published:** 2024-05-14

**Authors:** Xue-Yan Zhang, Bo-Chi Zhu, Miao He, Shan-Shan Dong

**Affiliations:** 1 School of Nursing, Jilin University, Changchun, 130021 Jilin China; 2 Department of Neurology, Second Hospital of Jilin University, Changchun, 130022 Jilin China; 3 Department of Anesthesiology, Second Hospital of Jilin University, No. 218 Ziqiang Street, Changchun, 130022 Jilin China

**Keywords:** c-Myb, NKILA, LIN28A, Let-7, Cisplatin resistance, Ovarian cancer

## Abstract

Ovarian cancer is a major gynecological cancer that has poor prognosis associated mainly to its late diagnosis. Cisplatin is an FDA approved ovarian cancer therapy and even though the therapy is initially promising, the patients mostly progress to resistance against cisplatin. The underlying mechanisms are complex and not very clearly understood. Using two different paired cell lines representing cisplatin-sensitive and the cisplatin-resistant ovarian cancer cells, the ES2 and the A2780 parental and cisplatin-resistant cells, we show an elevated proto-oncogene c-Myb in resistant cells. We further show down-regulated lncRNA NKILA in resistant cells with its de-repression in resistant cells when c-Myb is silenced. NKILA negatively correlates with cancer cell and invasion but has no effect on cellular proliferation or cell cycle. C-Myb activates NF-κB signaling which is inhibited by NKILA. The cisplatin resistant cells are also marked by upregulated stem cell markers, particularly LIN28A and OCT4, and downregulated LIN28A-targeted *let-7* family miRNAs. Whereas LIN28A and downregulated *let-7*s individually de-repress c-Myb-mediated cisplatin resistance, the ectopic expression of *let-7*s attenuates LIN28A effects, thus underlying a c-Myb-NKILA-LIN28A-*let-7* axis in cisplatin resistance of ovarian cancer cells that needs to be further explored for therapeutic intervention.

**Supplementary Information:**

The online version contains supplementary material available at 10.1186/s13048-024-01429-w.

## Introduction

Ovarian cancer is a gynecological cancer that is marked by diagnosis in late stages which relates with its poor outcomes [1]. In big population countries such as China, the burden of ovarian cancer is enormous, with close to 200,000 cases and 29000 deaths every year [2]. In developed countries such as the US, ovarian cancer ranks fifth in cancer deaths among women with a lifetime chance of getting this cancer being 1 in 78 [3]. An even more disturbing fact is that there has been an obvious upward trend in ovarian cancer statistics with a significant acceleration in last five years [2]. According to US National Cancer Institute, the 5-year relative survival rate for all types of ovarian cancer is 49.1% and it is estimated that more than three-fourths of ovarian cancer patients are diagnosed at an advanced stage, primarily owing to the disease being asymptomatic at early stages [4]. The burden of ovarian cancer and the associated deaths are predicted to rise for the forthcoming many years, which owing to late diagnosis poses specific challenges in the overall management resulting in increased mortality, and that’s why it is extremely important to tackle this problem.

There are several treatment options for ovarian cancer patients. These include monotherapies such as cisplatin, docetaxel, etoposide, gemcitabine, topotecan as well as combinational therapies such as carboplatin + paclitaxel. Among these, cisplatin is a prominent chemotherapy. It is a non-specific anticancer therapy that interferes with DNA synthesis thereby halting tumor growth [5]. Cisplatin crosslinks with purine bases, disrupts DNA repair mechanisms and induces apoptosis [6]. It further induces DNA damage and impacts the integrity of nucleic acids by forming cisplatin-DNA adducts, induction of oxidative stress and inducing mitochondrial damage [7]. Since cisplatin has been considered an effective way of treating ovarian cancer for many decades, its use has been widespread [8]. However, with the widespread use of cisplatin in ovarian cancer patients, the observation of cisplatin resistance in ovarian cancer patients has also been made [9–11]. Whereas up to 70% ovarian cancer patients initially respond well to cisplatin [12], it is believed that almost half of these patients eventually stop responding and develop resistance, that is marked by disease progression and poor prognosis. In an effort to overcome this phenomenon of cisplatin resistance in ovarian cancer patients, often a combinational approach is followed, wherein cisplatin is administered in combination with other drugs such as paclitaxel (taxol), carboplatin or docetaxel [7]. Additionally, combination of cisplatin with several other potential anticancer agents of dietary origin has also been evaluated [7, 13]. As suggested by numerous research activities and publications on the subject, a number of mechanisms have been implicated in cisplatin resistance of ovarian cancer, including a role of non-coding RNAs, particularly the long non-coding RNAs (lncRNAs). In recent years, lncRNAs have gathered a lot of attention for their influence on cisplatin sensitivity and resistance. LncRNAs belong to the class of non-coding RNAs – they are relatively longer (more than 200 nucleotides long) and do not code for any protein products. lncRNAs are increasingly being implicated in tumorigenesis, including resistance against therapies [14–16]. Of the many lncRNAs found to play a role in chemo-resistance of ovarian cancer cells, including cisplatin resistance, some prominent ones are MALAT1 [17–19], HOTAIR [20–22], ANRIL [23, 24]. Based on the emerging data, it is evident that lncRNAs are potential targets to overcome cisplatin resistance in ovarian cancer [19, 25].

C-Myb is a proto-oncogene [26] and a transcription factor [27] that has been implicated in several cancers where it promotes resistance [28, 29]. One earlier report has even suggested a role of c-Myb in ovarian cancer cisplatin resistance [30] but not much is known beyond this only report. In this study, we first evaluated a role of c-Myb in cisplatin resistance of ovarian cancer using two paired cell lines and then we set out to elucidate a mechanism. We report a novel lncRNA NKILA for its role in the c-Myb mediated cisplatin resistance. We also show a mechanism that includes enrichment of stem cells, particularly the stem cell marker LIN28A which then targets and represses *let-7* family microRNAs (miRNAs).

## Materials and Methods

### Cell lines and other materials

ES2 cells were purchased from ATCC (Manassas, USA) and cultured in McCoy’s 5a medium with 10% FBS in 5% CO_2_–humidified incubators at 37°C. siRNA against c-myb was purchased from SCBT (USA). Cisplatin resistant ES2 cells were generated in the laboratory by long term exposure (over four months) of the cells to cisplatin with gradual increase of cisplatin concentration after every 4-5 passages (Supplementary Figure S1). Parental and cisplatin resistant A2780 cells were purchased from Sigma (St Louis, USA) and cultured in RPMI 1640 media with 10% FBS in 5% CO_2_–humidified incubators at 37°C. For the routine maintenance and propagation, cisplatin resistant cells were cultured with sub-IC-50 concentrations of cisplatin in the culture media and the cisplatin resistance of these cells was periodically confirmed by evaluating the IC-50 values.

### Transfections

All transfections, such as anti-*let-7*s and pre-let-7s (let-7d/e/f) were performed in six wells plates. 4.5 x 10^5^ cells were seeded overnight. They were then transfected with pre-let-7s/anti-let-7s or miRNA-negative controls (Ambion, China) at a final concentration of 20 nM, using DharmaFECT1 transfection reagent (Dharmacon, China). After 48-72 hours, cells were collected again by trypsinization, counted, re-seeded in 6-well plates and pre/anti-miRs or the negative controls were added for two more rounds of transfections of 72 hours each.

### NKILA downregulation

We downregulated NKILA using locked nucleic acid GapmeR from Qiagen (China). For control conditions, a control LNA GapmeR was used. Cells were transfected at ~60-70% confluency with 20 nM LNA GapmeRs, using Lipofectamine RNAiMax (Thermo Fisher Scientific, China), as reported by others [31].

### C-Myb detection

c-Myb ELISA kit (LifeSpan BioSciences, Inc., China) was used for the detection of c-Myb levels. This assay uses sandwich ELISA method wherein each well of the supplied microtiter plate comes pre-coated with a target specific capture antibody. Standards or samples (100μl) were added to the wells and incubated at 37^0^C for 1 h for the target antigen to bind to the anti-c-Myb capture antibody. 100μl of supplied biotin-conjugated detection antibody was added and gently agitated at 37^0^C for 1 h. This was followed by 3 times washing with wash buffer. 100μl of avidin-horseradish peroxidase (HRP) conjugate was added for 1 h at room temperature and then washed 5 times before addition of 90μl of TMB substrate for 30 minutes. Stop solution consisting of sulfuric acid was added to terminate color development reaction and optical density (OD) was measured at a wavelength of 450 nm, using Shimadzu reader.

### BrdU cell proliferation assay

BrdU (5-bromo-2’-deoxyuridine) method was used to study cell proliferation. The kist was purchased from Cell Signaling (China). This assay detects BrdU that is incorporated in the cellular DNA during cell proliferation, using an anti-BrdU antibody. 4000 cells are cultured in individual wells of 96-well plates with labeling medium that contained BrdU and this pyrimidine analog replaced thymidine into the newly synthesized DNA of proliferating cells. At the end of incubation time (72 hours), labeling medium was removed and 100μl of fixing/denaturation solution was added at room temperature for 30 minutes. This was followed by addition of 1X detection antibody in a total volume of 100μl at room temperature for 1 h. Then the plate was washed 3 times with supplied wash buffer before addition of anti-mouse IgG, HRP-linked antibody to recognize the bound detection antibody. 100μl HRP substrate TMB was added to develop color which was read at 450nM, using Shimadzu reader.

### Colony-formation assay

Anchorage-dependent colony formation assay was performed to assess colony forming ability of ovarian cancer cells. Cells were collected by trypsinization and resuspended in complete culture medium. Single cell suspensions were seeded in 6-well plates at a density of 1000 cells per well overnight and then suitably treated. After three weeks of growth in an incubator under 5% O_2_, 5% CO_2_ and 90% N_2_ conditions, colonies were fixed in 4% paraformaldehyde, and then stained with crystal violet. Pictures were taken and the colonies were calculated, using NIH Scion image analysis software.

### Cell invasion assay

We performed cell invasion assay using 24-well plates with inserts (8 µM pores). Inserts were coated with growth factor reduced Matrigel. Single cell suspension of cells was first obtained and then the cells were onto the inserts with medium without FBS. The bottom of the wells contained medium with FBS that acted as attractant for invasion. Cells that invaded through matrigel were stained using 4 µg/ml Calcein AM (ThermoFisher Scientific, China) in PBS for an hour at room temperature and cells were recovered from the bottom of inserts by trypsinization and counted, using hemocytometer. Also, of invaded cells was quantitated by collecting all invaded cells from individual test conditions into individual wells of a 96-well plate and reading fluorescence using a fluorescence plate reader.

### ELISA for NF-κB and STAT3

ELISA kits were purchased from Abcam (China) to detect activation of NF-κB and STAT3. The individual assays for NF-κB as well as STAT3 detect phosphorylated as well as total factor in a single assay. The NF-κB kit detected total NF-κB as well as phospho- NF-κB-p65 S468+S536. The STAT3 kit detected total STAT3 as well as phospho-STAT3 Y705. Absorbance at 450nM was read using a Shimadzu plate reader.

### Quantitative RT-PCR for detection of NKILA and *let-7* miRNAs

The primers and reagents for the detection of NKILA and *let-7*s were purchased from Qiagen (China). RT2 first strand kit (Qiagen, China) was used for cDNA synthesis. To 1μg of RNA, 2μl of genomic DNA elimination mix was added and mixed, incubated for 5 minutes at 420C and then quickly transferred to ice-cold water for 1 minute. Reverse transcription mix (5x buffer with reverse transcriptase enzyme) was then added and incubated for 15 minutes at 42^0^C. Reaction was stopped by heating the mixture to a temperature of 95^0^C. All lncRNAs were detected using probes from Qiagen (China). qPCR for miRNAs was conducted using probes and primers from Thermo Scientific Fisher (China) according to manufacturer’s instructions. Results were normalized using glyceraldehyde-3-phosphate dehydrogenase (GAPDH) or U6 as an internal control.

### In vivo experiments

Our *in vivo* experiments were approved by the Animal Research Ethics Committee at the Jilin University. All methods were performed in accordance with the relevant guidelines and regulations. 1 million ES2/ES2C/ES2C-si cells were injected subcutaneously into both flanks of female ICR-NOD/SCID mice (Vital River Laboratories, Co., Ltd., China). *N*=6 mice were included in all experimental groups and the mice were housed in sterilized room with food and water provided *ad libitum*. The three groups of mice were– Group 1: injected with ES2 cells (ES2); Group 2: injected with ES2C cells (ES2C) and Group 3: injected with ES2C cells silenced for c-Myb (ES2C-si) Tumors were allowed to proliferate for 5 weeks before the sacrifice. Tumors were measured using calipers and the volume of tumors in mm^3^ was determined by the formula (width^2^ x length)/2.

### Statistical considerations

The results reported here are representative from at least three repeats. Unpaired t-test and one way ANOVA was used, as appropriate, to calculate *p* values and determined significance. *p* value < 0.05 was considered statistically significant.

## Results

### c-Myb and cisplatin resistance

**Fig. 1:**
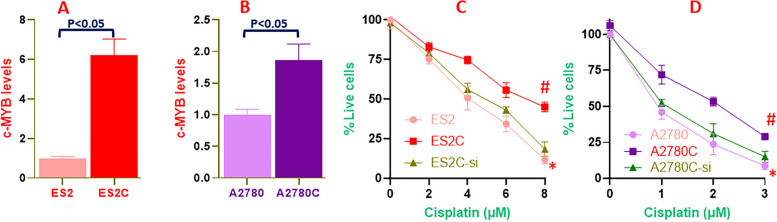
C-Myb and cisplatin resistance. C-Myb levels were detected in parental and cisplatin resistant ES2 (**A**) and A2780 (**B**) cells. Cell proliferation, to assess cytotoxic effects of increasing cisplatin, was assessed in parental and cisplatin resistant ES2 (**C**) and A2780 (**D**) cells with an additional silencing of c-Myb in resistant cells (ES2C-si / A2780C-si). The presented results are representative of at least 3 different repeats with triplicate samples in each repeat. #*p*<0.05, compared to control. *p<0.05, compared to resistant cells without silencing of c-Myb.

Our investigation into the mechanism of cisplatin resistance of ovarian cancer cells started with a characterization and establishment of appropriate cell model system. To ensure confidence in results, we decided to choose a minimum of two ovarian cancer cell lines. Also, in order to study cisplatin resistance, we evaluated paired cell lines – parental cells and their cisplatin-resistance derivatives. We first looked at c-Myb levels in parental vs. cisplatin resistant cells and observed that cisplatin-resistance ES2 cells (ES2C) had much higher c-Myb levels when compared to the parental ES2 cells (ES2) (Fig. 1A). the levels of c-Myb were more than six-folds higher in ES2C cells (*p*<0.05). To further check the involvement of c-Myb in cisplatin resistance of ovarian cancer cells, we confirmed the finding in another paired cell lines and observed similar results i.e. c-Myb was significantly higher in the cisplatin-resistance A2780 cells (A2780C) when compared to the parental A2780 cells (A2780) (Fig. 1B).

Since both of the paired cell lines exhibited higher c-Myb levels in cisplatin resistant cells, we hypothesized that c-Myb is probably involved in cisplatin resistance of ovarian cancer cells. To confirm this, we conducted BrdU proliferation assay and observed that in paired ES2 cells, ES2C cells resisted killing against cisplatin when the cells were subjected to cisplatin treatment for 72 hours (Fig. 1C). When c-Myb was silenced in the cisplatin resistant ES2C cells (ES2C-si), the cells became much more sensitive to cisplatin and behave similar to the parental ES2 cells. This proved that c-Myb indeed played a role in cisplatin resistance of ES2 cells. When we performed similar experiment in paired A2780 cells, we observed similar trend. The cisplatin resistant A2780C cells were much more resistant against cell killing by cisplatin (Fig. 1D). However, when c-Myb was silenced in resistant cells (A2780C-si), the resistance was gone and the cells became much more sensitive to cisplatin, similar to the parental cells. The IC-50 value of ES2C cells, relative to parental ES2 cells, was found to be increased 87.8% while that of A2780C cells, relative to parental A2780 cells, was found to be increased 172.6% (Table 1).

**Table 1:** IC-50 values of ovarian cancer cell lines

**Cell Line**	**IC-50 (μM)**	**% increase **^**a**^
ES2	4.11 ± 0.18	-
ES2C	7.72 ± 0.24	87.8%
A2780	0.84 ± 0.04	-
A2780C	2.29 ± 0.11	172.6%

^a^% increase in IC-50 values in cisplatin-resistant cells, relative to respective parental cells

The experiment was repeated four different times and representative values are presented. Each of the four individual assays had triplicate repeats in each assay

### LncRNAs involvement in cisplatin resistance

**Fig. 2:**
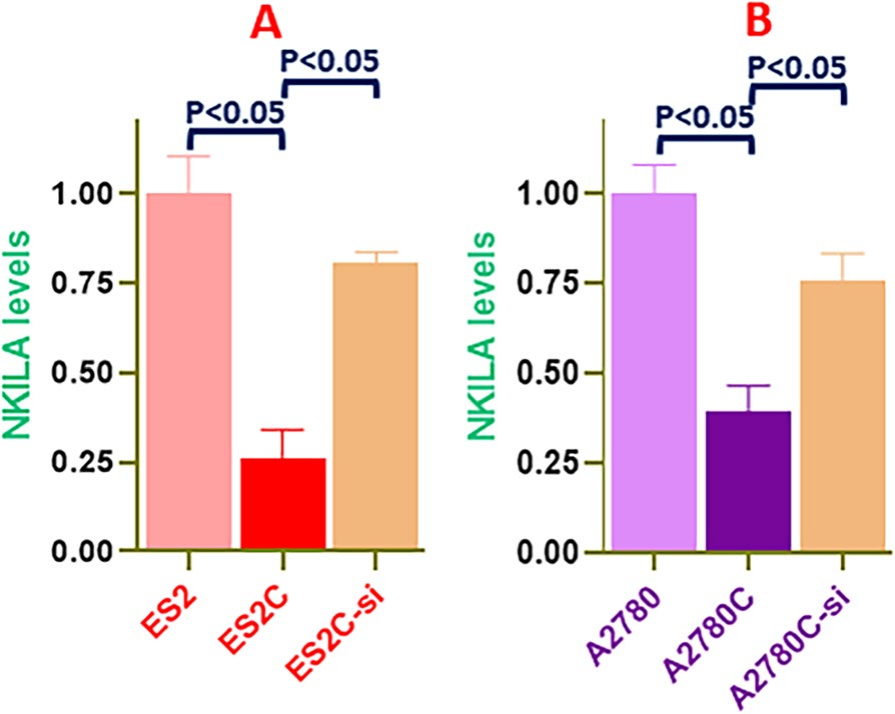
LncRNA NKILA negatively associates with cisplatin resistance and c-Myb expression. qRT-PCR was used to assess NKILA levels in parental and cisplatin resistant ES2 (**A**) and A2780 (**B**) cells with an additional silencing of c-Myb in resistant cells (ES2C-si / A2780C-si). The presented results are representative of at least 3 different repeats with triplicate samples in each repeat.

A number of reports have provided evidence for a role of lncRNAs in drug resistance of cancer cells [32–34] thus providing a rationale for the suitability of these non-coding RNAs in differentiating between cisplatin resistance vs cisplatin sensitivity [35]. We screened a number of lncRNAs for their differential expression in cisplatin resistant vs cisplatin sensitive ovarian cancer cells. Our initial screening was in ES2C vs ES2 cells and only lncRNAs that were differentially expressed in this model were further checked in the second model system comprising of A2780C vs A2780 cells. Based on this screening, a number of lncRNAs were found to be differentially expressed in cisplatin resistant vs. cisplatin sensitive ovarian cancer cells (Supplementary Figure 1). However, the lncRNA that stood out was NKILA. As shown in Fig. 2A, NKILA levels were significantly lower in ES2C cells, compared to the parental ES2 cells, which suggested that this lncRNA is a tumor suppressor lncRNA and relates inversely with cisplatin resistance. To ascertain its role in c-Myb signaling, we tested its levels in ES2C-si cells and found that NKILA was much highly expressed in these cells (Fig. 2A) which suggested that NKILA levels inversely related with c-Myb levels as well. We further confirmed this in A2780 paired model and observed that while NKILA was downregulated in resistance A2780C cells, the expression went up in A2780C-si i.e. when C-Myb was silenced (Fig. 2B). Combined, these results established an inverse relationship between NKILA and c-Myb/cisplatin resistance.

### NKILA effects on cancer cell properties

**Fig. 3:**
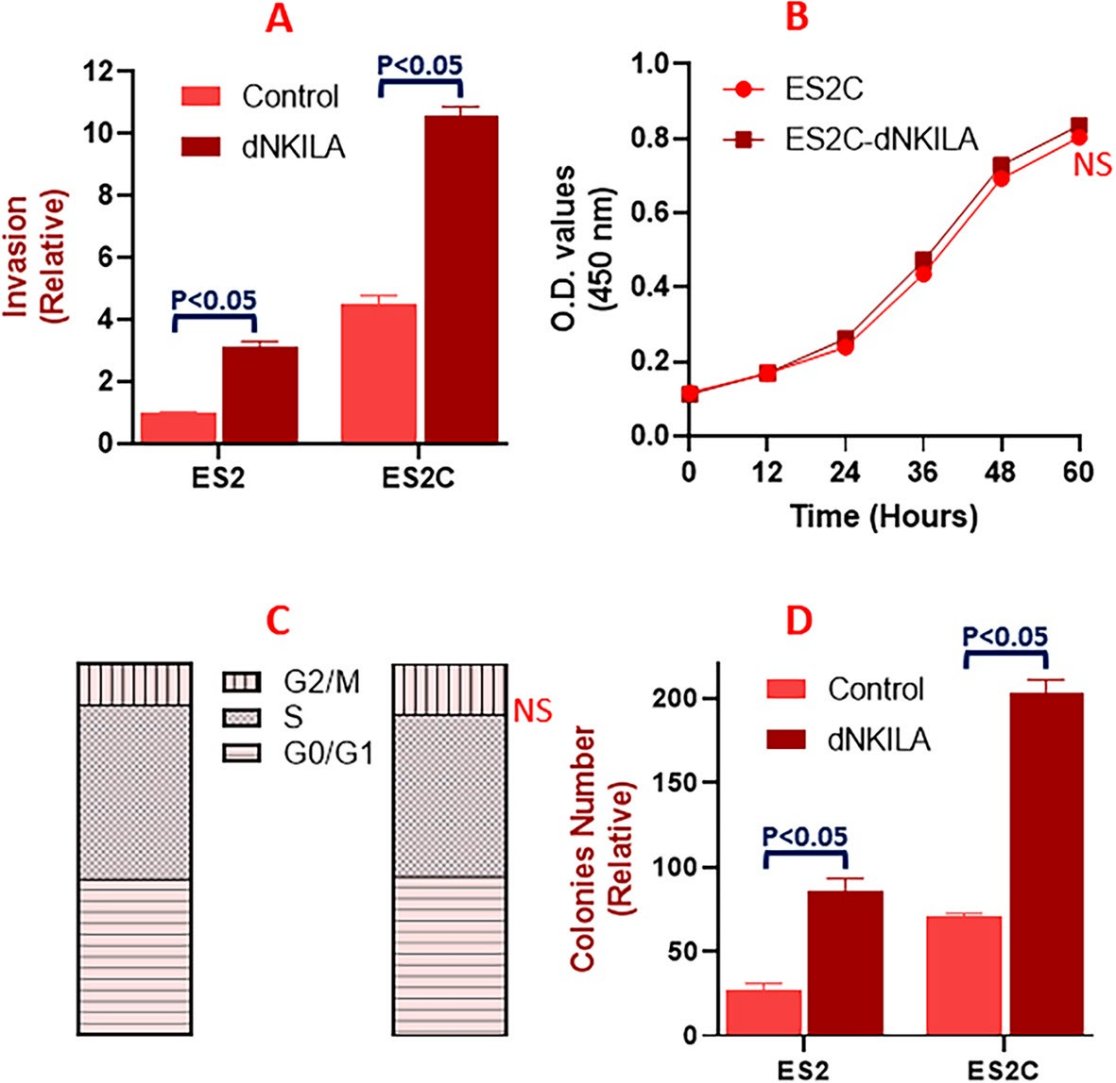
Effect of NKILA downregulation on cell characteristics. Invasion (**A**), proliferation (**B**), cell cycle (**C**) and colony forming ability (**D**) was assessed in parental and cisplatin resistant ES2 cells. The presented results are representative of at least 3 different repeats with triplicate samples in each repeat. NS: non-significant results

Having observed a possible role of NKILA in c-Myb signaling and cisplatin resistance of ovarian cancer cells, we next evaluated if NKILA could play a role in different characteristics associated with cancer cells. For this, we first checked the effect on NKILA on invasion potential by downregulating NKILA in both parental and cisplatin resistant cells. We observed that in parental ES2 as well as cisplatin resistant ES2C cells, downregulation of NKILA led to significant increase in the invasion potential (Fig. 3A). As can be seen, the invasion of cisplatin resistant cells was already more than the parental cells, as expected, and this was further potentiated by downregulation of NKILA. Cancer cells are usually highly proliferating cells and we checked the effect of NKILA on cell proliferation. Surprisingly, as shown in Fig. 3B, we did not see much effect of downregulation of NKILA in ES2C cells, which meant that probably NKILA does not contribute to cell proliferation. Similarly, little to no effect of NKILA downregulation was observed on the cell cycle of ES2C cells (Fig. 3C). However, when we checked the colony forming ability, we found a significant effect of NKILA. The number of colonies formed by the same number of starting cells were much higher in cisplatin resistant cells and moreover downregulation of NKILA increased these colonies by multiple folds (Fig. 3D). Combined, the results showed that whereas NKILA affects invasion and colony forming ability of ovarian cancer cells, it does not seem to have any effect on cell proliferation or cell cycle progression.

### C-Myb / NKILA effects on NF-κB signaling

**Fig. 4:**
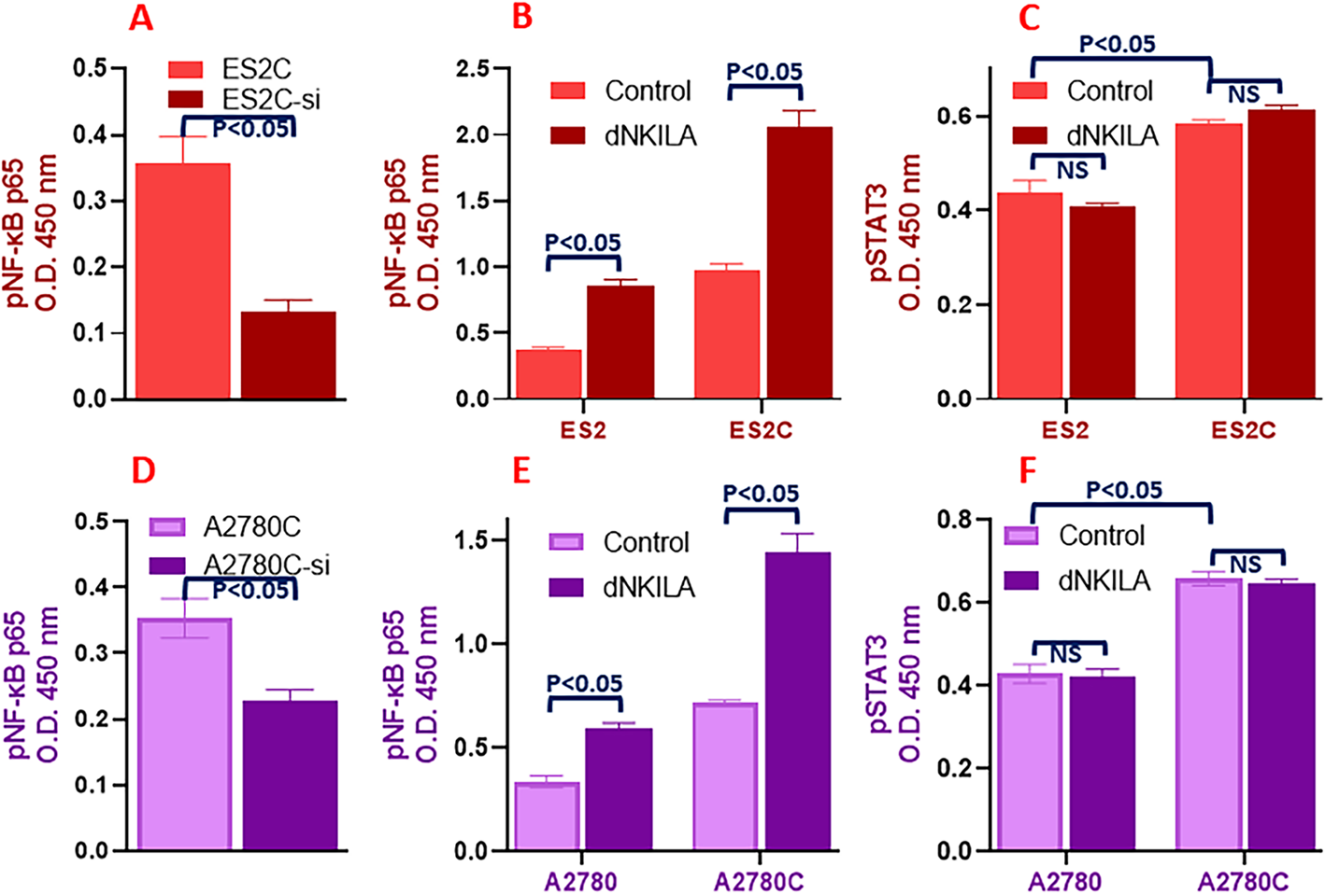
NF-κB activation in cisplatin resistant cells and the effect of lncRNA NKILA. (**A**) NF-κB was evaluated in resistant ES2 cells with (ES2C-si) and without (ES2C) c-Myb silencing. Effect of downregulation of NKILA on NF-κB activation (**B**) and STAT3 activation (**C**) was assessed in parental and cisplatin resistant ES2 cells. (**D**) NF-κB was further evaluated in resistant A2780 cells with (A2780C-si) and without (A2780C) c-Myb silencing. Effect of downregulation of NKILA on NF-κB activation (**E**) and STAT3 activation (**F**) was also assessed in parental and cisplatin resistant A2780 cells. The presented results are representative of at least 3 different repeats with triplicate samples in each repeat. NS: non-significant results

Since NKILA is a lncRNA that interacts with, and possibly modulates NF-κB signaling [36], we became interested in evaluating NF-κB signaling in the cells with varying c-Myb and NKILA levels. In the ES2C cells with c-Myb silenced, we observed a significantly reduced NF-κB signaling (Fig. 4A), when compared with the NF-κB signaling in the ES2C cells without c-Myb silenced. Further, in ES2 as well as ES2C cells, downregulation of NKILA (dNKILA) led to significant increase in activation of NF-κB (Fig. 4B). We also tested for the ability of NKILA to further activate STAT signaling. However, we observed that NKILA had no effect on STAT3 activation in ES2 or ES2C cells (Fig. 4C). Our findings were further confirmed in A2780 cells. First, we observed lower NF-κB activation in A2780C cells that were silenced for c-Myb (Fig. 4D) and then whereas both NF-κB and STAT3 were activated in cisplatin resistant A2780C cells, compared to parental A2780 cells, NKILA downregulation could only induce NF-κB signaling (Fig. 4E) but not STAT3 signaling (Fig. 4F).

### Effects on cancer stem cell markers and miRNAs

**Fig. 5:**
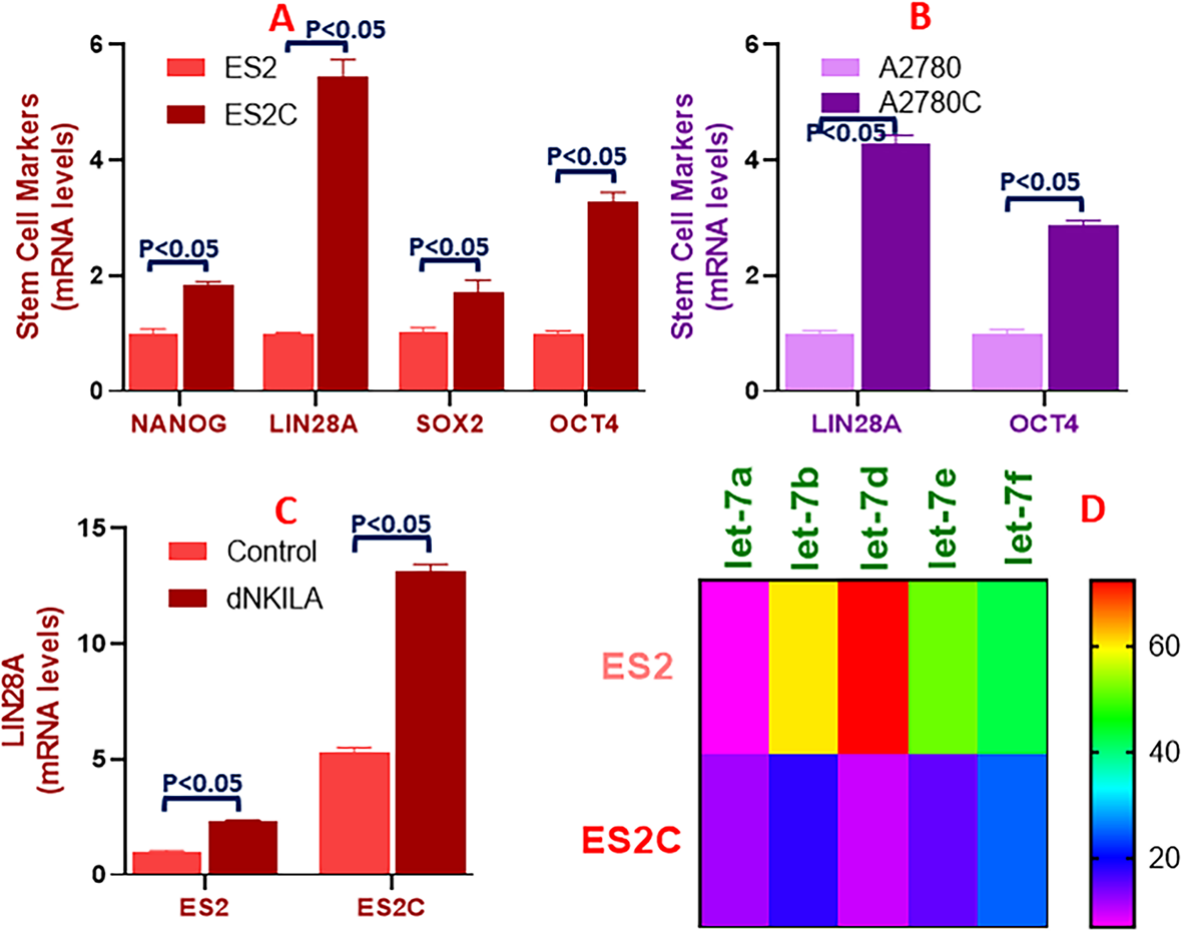
The lncRNA-stem cells-miRNAs axis. qRT-PCR was conducted to assess mRNA levels of stem cells markers in parental and cisplatin resistant ES2 (**A**) and in parental and cisplatin resistant A2780 (**B**) cells. **C** mRNA levels of stem cells marker LIN28A were assessed in parental and cisplatin resistant ES2 cells with (dNKILA) and without (Control) NKILA downregulation. **D** qRT-PCR was used to assess levels of let-family miRNAs in parental and cisplatin resistant ES2 cells. The presented results are representative of at least 3 different repeats with triplicate samples in each repeat

c-Myb is known to influence cancer stem cell characteristics and we next hypothesized that the cisplatin resistance of ovarian cancer cells also involves dysregulated cancer stem cell characteristics. To test this hypothesis, we first evaluated levels of stem cell biomarkers in ES2C vs ES2 cells. It was observed that all the stem cell markers tested were expressed at higher levels in the cisplatin resistant cells (Fig. 5A). The stem cell marker with highest levels was LIN28A which was expressed at more then five-folds higher level in ES2C cells (Fig. 5A). OCT4 was the next most elevated stem cell marker. To confirm these results, particularly to verify the two most elevated stem cell markers LIN28A and OCT4, we checked A2780C vs. A2780 cells and observed a similar elevated levels of both of these stem cell markers in the cisplatin resistant cells in this paired group of cell lines as well (Fig. 5B). We next checked if lncRNA NKILA would have an effect on LIN28A and we observed that downregulation of NKILA in both ES2 as well as ES2C cells resulted in significantly elevated levels of LIN28A (Fig. 5C). lncRNAs mostly function via sponging of microRNAs (miRNAs) and moreover LIN28A is known to target *let-7* family of miRNAs, therefore, we also checked the levels of several *let-7* family miRNAs in ES2C vs ES2 cells. We observed dysregulation of several *let-7* family miRNAs with *let-7d* being the most significantly downregulated miRNA in the cisplatin resistant cells with *let-7e* and *let-7f* also significantly downregulated and the *let-7b* moderately downregulated (Fig. 5D).

### c-Myb-lncRNA-miRNA-stem cell axis in cisplatin resistance

**Fig. 6:**
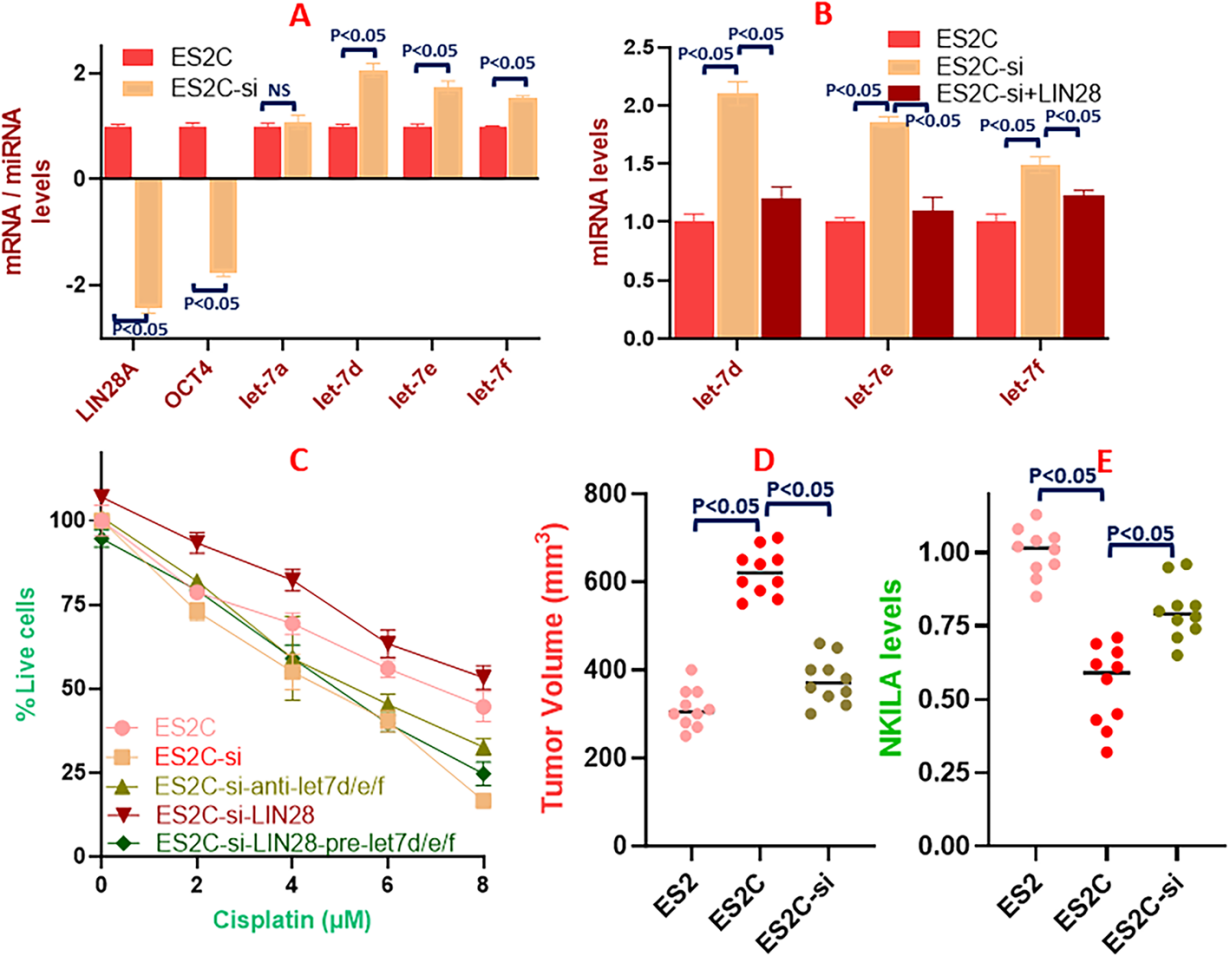
c-Myb-NKILA-LIN28A-miRNAs axis in cisplatin resistance. **A** qRT-PCR was conducted to assess mRNA levels of stem cells markers and let-7 family miRNAs in cisplatin resistant ES2 cells with (ES2C-si) and without (ES2C) c-Myb silencing. **B** qRT-PCR was conducted to assess let-7 miRNAs in cisplatin resistant ES2 cells with (ES2C-si) and without (ES2C) c-Myb silencing, along with overexpressed LIN28 in c-Myb silenced cells (ES2C-si+LIN28A). **C** Cell proliferation, to assess cytotoxic effects of increasing cisplatin, was assessed in cisplatin resistant ES2 cells with (ES2C-si) and without (ES2C) c-Myb silencing, along with few more conditions such as anti-let-7s in c-Myb silenced cells (ES2C-si-antiletd/e/f) overexpressed LIN28 in c-Myb silenced cells (ES2C-si-LIN28A) and additional pre-let7s in c-Myb silenced and LIN28 overexpressing cells (ES2C-si-LIN28A-pre-let7d/e/f). The presented results are representative of at least 3 different repeats with triplicate samples in each repeat. **D** Tumor volumes were measured in mice implanted with 1 million ES2, ES2C and ES2C-si cells and after tumor was allowed to progress for five weeks. **E** qRT-PCR was used to quantitate the levels of lncRNA NKILA in the tumor remnants from Figure 6D. NS: non-significant results

After the many findings as detailed above, we finally confirmed the mechanistic relationship between c-Myb, lncRNA NKILA, miRNAs and the stem cell marker LIN28A leading to cisplatin resistance. First, we confirmed the relationship between c-Myb and LIN28A.miRNAs. When we compared the expression levels in ES2C cells vs. ES2C cells with silenced c-Myb, stem cell marker LIN28A was downregulated more than 2-folds; OCT4 was also significantly downregulated (Fig. 6A). However, all the *let-7* family miRNAs, with the exception of *let-7a*, were significantly upregulated (Fig. 6A). To establish the mechanism of LIN28A-regulated *let-7* family miRNAs in c-Myb mediated cisplatin resistance, we further transfected LIN28A in the ES2C cells with silenced c-Myb. Such overexpression of LIN28A resulted in repression of *let-7d*, *let-7e* as well as *let-7f* (Fig. 6B) thus verifying that LIN28A-reguation of *let-7* family miRNAs plays a role in c-Myb signaling as well as the resulting cisplatin resistance. As a direct effect on cisplatin resistance, we observed that in ES2Ccells with silenced c-Myb, antagonizing *let-7d/e/f* overcame the repressing effects of c-Myb silencing and the cells were once again resistant to cisplatin (Fig. 6C). When instead of antagonizing *let-7*s, if LIN28A was overexpressed, the results were similar and even more significant with the resistance against cisplatin even more pronounced. Moreover, when *let-7*s were added to this LIN28A transfected and c-Myb silenced ES2C cells, the cells yet again became sensitive to cisplatin (Fig. 6C).

### In vivo validation

Finally, to verify that the observations could be tested and established *in vivo*, we used mouse experiment wherein a million cells (ES2, ES2C and ES2C-si) were injected into two flanks of *n*=6 mice in each group. As seen in Fig. 6D, when the tumors were allowed to form, there was a considerable difference in the tumor size at the end of 5 weeks. The tumors caused by cisplatin resistant ES2C cells were significantly larger than those by parental ES2 cells (Fig. 6D). The average tumor size in ES2C mice (622 mm^3^) was almost double (1.98 folds), as compared to the average tumor size in ES2 mice (313 mm^3^) (*p*<0.05). At the same time the levels of lncRNA NKILA were significantly reduced in ES2C cells, as compared to the ES2 cells (Fig. 6E). The downregulation of NKILA across the tumors in ES2C mice was found to be -1.83 folds (*p*<0.05), as compared to average NKILA levels in ES2 mice (Fig. 6E). Further, we found that, as compared to ES2C cells, the ES2C cells with c-Myb silenced (ES2C-si) formed relatively smaller tumors (Fig. 6D) which supported the *in vitro* findings. The average tumor size in ES2C-si group was 376 mm^3^, which was -1.64 folds reduction in tumor size, when compared to ES2C group without the silencing of c-Myb (*p*<0.05) (Fig. 6D). Also, the NKILA levels were de-repressed in these tumors (Fig. 6E) and we found that c-Myb silencing increased NKILA expression by 1.46 folds in the ES2C-si mice, when compared to the mice without c-Myb silencing, i.e. ES2C mice (*p*<0.05) (Fig. 6E). These *in vivo* results clearly support our overall findings that c-Myb drives tumorigenesis and cisplatin resistance and that c-Myb and NKILA levels are inversely correlated.

## Discussion

The emergence of cisplatin resistance in ovarian cancer patients is a major setback in the treatment of ovarian cancer. A number of underlying mechanisms have been proposed but a clear understanding is lacking. In the present study, we used two different paired cell lines to study cisplatin resistance in ovarian cancer. One system used was the paired A2780 model which was obtained commercially. It comprised of cisplatin sensitive as well as cisplatin resistant A2780 cells. Additionally, we developed another paired model in our laboratory comprising of ES2 cells. To accomplish this, we cultured cisplatin sensitive parental cells in the presence of cisplatin for long time that resulted in acquisition of cisplatin resistance. Thus, we employed a commercially available as well as an in-house model for cisplatin resistance. This ensured confidence in our findings and moreover, we observed that the mechanism was very similar in both paired models, thus validating our findings.

Our initial hypothesis comprised of a possible role of transcription factor and proto-oncogene c-Myb in cisplatin resistance of ovarian cancer cells. In one of the early reports on the subject, a role of c-Myb in cisplatin resistance was reported in colon cancer cells [37]. This study focused on colon cancer and it was shown that antagonizing c-Myb could sensitize colon cancer cells to cisplatin. There also are two reports on c-Myb’s activity in cisplatin resistance of ovarian cancer cells. In one such report, C-Myb was reported to induce cisplatin resistance by activating NF-κB and STAT-3 signaling [38]. The focus of this study was on a modulatory effect of this c-Myb mediated action by dietary constituents. In the other report on c-Myb mediated ovarian cancer cisplatin resistance, an miRNA mediated mechanism was reported [30]. C-Myb was reported to induce oncogenic miRNA miR-21 which correlated with increased tumor growth *in vivo*.

A number of reports have helped establish a possible role of lncRNAs in drug resistance of ovarian cancer, including cisplatin resistance [21, 31, 39–44]. Based on these emerging evidence, it can be safely concluded that lncRNAs are valid therapeutic targets to overcome the chemoresistance in ovarian cancer [25]. We provide first evidence for the role of lncRNA NKILA in cisplatin resistance of ovarian cancer. Such specific role of this lncRNA has never been reported even though NKILA has been implicated in resistance mechanism in other cancers [45]. Of note, NF-κB signaling has been implicated in ovarian cancer cisplatin resistance [38, 46, 47] as also corroborated in results shown here. Thus, whereas a role of NF-κB signaling is evident in ovarian cancer cisplatin resistance, further mechanisms remain unknown. Through this study of ours, particularly from our observations with the lncRNA NKILA which targets NF-κB, we provide a rationale and mechanism for this reported and established involvement of NF-kB in cisplatin resistance. In our observations, we noted that NKILA affected invasion and colonies but not proliferation or cell cycle. Of note, we observed higher NF-κB activation in cisplatin resistant cells (both ES2C and A2780C cells), as compared to the respective parental cells (ES2 and A2780 cells), which is in agreement with earlier reports of increased NF-κB signaling in cisplatin resistance ovarian cancer cells. The further observation of an increased NF-κB signaling in cells with downregulated NKILA confirms the NF-κB-targeting activity of lncRNA NKILA as well as its role in cisplatin resistance of ovarian cancer cells. We also tested STAT3 activation as STAT3 has also been shown to be activated in cisplatin resistant ovarian cancer cells [38]. However, this was done not to ascertain a role of STAT3 activation in cisplatin resistance but rather to study the specificity of NKILA action. Our results showing an increased STAT3 phosphorylation in cisplatin resistant, compared to parental cells, are clear proof for STAT3 activation in cisplatin resistance even in our model system. However, the failure of NKILA to have any impact on this STAT3 activation is a clear proof that the NKILA action is only on NF-κB, and not on STAT3 signaling. It also needs to be pointed out that the robust delivery systems for lncRNAs are still being tested but our study does provide an indication that when appropriate methodology is developed, lncRNA NKILA can be delivered to ovarian patients to overcome cisplatin resistance.

The role of c-Myb in determining cancer stem cell characteristics is still not very clear. While there is evidence for its possible repression of cancer stem cell characteristics in lung cancer leading to inhibition of cancer metastasis [48], there is also evidence for its regulation of cancer stem cell characteristics and the hierarchy of stem-like T cells [49]. It positively regulates invasion of cancer cells as reported earlier [50] as well as shown in this study by us. Moreover, we show an upregulation of several cancer stem markers in cisplatin resistance cells, which is in general agreement with the published literature on a role of cancer stemness in cisplatin resistance [51] and we also show a particular positive correlation between cancer stem marker LIN28A with cisplatin resistance, along with a negative correlation of LIN28A with the lncRNA NKILA. In our study we found LIN28A to be the most highly expressed stem cell marker in cisplatin resistant cells with OCT4 as the next elevated stem cell marker. This might be due to the direct positive regulation of OCT4 by LIN28A, as has been reported previously [52].

LncRNAs are known to function through their targeting and sponging of miRNAs. In our study we did not search for an unbiased list of miRNAs that could be affected by upregulated stem cell biomarker. Based on the available knowledge that LIN28A targets *let-7* family, we focused on *let-7* family miRNAs. Our results indicated *let-7d* to be the most dysregulated miRNA in cisplatin resistant cells, followed by *let-7e* and *let-7f*, respectively. Interestingly, we did not observe much effect on *let-7a*, even though it has been shown to be regulated by LIN28A earlier [53]. This might be explained by the observation that LIN28A regulates biogenesis of all *let-7* miRNAs, except for *let-7a* [54]. Also, it is possible that *let-7a* might not be as relevant to cisplatin resistance of ovarian cancer cells, as the other of its family members.

In conclusion, in this study we provide a mechanism for C-Myb-mediated cisplatin resistance of ovarian cancer cells that involves downregulated lncRNA NKILA, activated NF-κB signaling, increased stemness marked by LIN28A and the resulting downregulated *let-7* family of miRNAs. This c-Myb-NKILA-LIN28A-*let7* axis represents a novel target for future therapy and management of cisplatin resistance ovarian cancers.

### Supplementary Information

Supplementary Material 1. 

## References

[1] Stuart GC, Kitchener H, Bacon M, duBois A, Friedlander M, Ledermann J, Marth C, Thigpen T, Trimble E, participants of 4th Ovarian Cancer Consensus C et al. 2010 Gynecologic Cancer InterGroup (GCIG) consensus statement on clinical trials in ovarian cancer: report from the Fourth Ovarian Cancer Consensus Conference. Int J Gynecol Cancer 2011, 21(4):750-755. 10.1097/IGC.0b013e31821b2568 21543936

[2] Wang Y, Wang Z, Zhang Z, Wang H, Peng J, Hong L (2023). Burden of ovarian cancer in China from 1990 to 2030: A systematic analysis and comparison with the global level. Front Public Health.

[3] Siegel RL, Miller KD, Wagle NS, Jemal A (2023). Cancer statistics, 2023. CA Cancer J Clin.

[4] Doubeni CA, Doubeni AR, Myers AE (2016). Diagnosis and management of ovarian cancer. Am Fam Phys.

[5] Song M, Cui M, Liu K (2022). Therapeutic strategies to overcome cisplatin resistance in ovarian cancer. Eur J Med Chem.

[6] Dasari S, Tchounwou PB (2014). Cisplatin in cancer therapy: molecular mechanisms of action. Eur J Pharmacol.

[7] Zon A, Bednarek I. Cisplatin in ovarian cancer treatment-known limitations in therapy force new solutions. Int J Mol Sci. 2023;24(8):7585. 10.3390/ijms24087585 PMC10146189 37108749

[8] Helm CW, States JC (2009). Enhancing the efficacy of cisplatin in ovarian cancer treatment - could arsenic have a role. J Ovarian Res.

[9] Li J, Feng Q, Kim JM, Schneiderman D, Liston P, Li M, Vanderhyden B, Faught W, Fung MF, Senterman M (2001). Human ovarian cancer and cisplatin resistance: possible role of inhibitor of apoptosis proteins. Endocrinology.

[10] Ortiz M, Wabel E, Mitchell K, Horibata S (2022). Mechanisms of chemotherapy resistance in ovarian cancer. Cancer Drug Resist.

[11] Marchetti C, De Felice F, Romito A, Iacobelli V, Sassu CM, Corrado G, Ricci C, Scambia G, Fagotti A (2021). Chemotherapy resistance in epithelial ovarian cancer: Mechanisms and emerging treatments. Semin Cancer Biol.

[12] Wang J, Wu GS (2014). Role of autophagy in cisplatin resistance in ovarian cancer cells. J Biol Chem.

[13] Minerva, Bhat A, Verma S, Chander G, Jamwal RS, Sharma B, Bhat A, Katyal T, Kumar R, Shah R (2023). Cisplatin-based combination therapy for cancer. J Cancer Res Ther.

[14] Wu Z, Jiang S, Chen Y (2024). Non-coding RNA and Drug resistance in cholangiocarcinoma. Noncoding RNA Res.

[15] Abdi E, Latifi-Navid S, Panahi A: Long noncoding RNA polymorphisms in gynecological cancers. Per Med. 2023;21(1):59–68. 10.2217/pme-2023-0082 38095072

[16] Usman M, Li A, Wu D, Qinyan Y, Yi LX, He G, Lu H (2024). The functional role of lncRNAs as ceRNAs in both ovarian processes and associated diseases. Noncoding RNA Res.

[17] Bai L, Wang A, Zhang Y, Xu X, Zhang X (2018). Knockdown of MALAT1 enhances chemosensitivity of ovarian cancer cells to cisplatin through inhibiting the Notch1 signaling pathway. Exp Cell Res.

[18] Wang Y, Wang X, Han L, Hu D (2020). LncRNA MALAT1 Regulates the Progression and Cisplatin Resistance of Ovarian Cancer Cells via Modulating miR-1271-5p/E2F5 Axis. Cancer Manag Res.

[19] Taheri M, Shoorei H, Tondro Anamag F, Ghafouri-Fard S, Dinger ME (2021). LncRNAs and miRNAs participate in determination of sensitivity of cancer cells to cisplatin. Exp Mol Pathol.

[20] Liu S, Lei H, Luo F, Li Y, Xie L (2018). The effect of lncRNA HOTAIR on chemoresistance of ovarian cancer through regulation of HOXA7. Biol Chem.

[21] Wang Y, Wang H, Song T, Zou Y, Jiang J, Fang L, Li P (2015). HOTAIR is a potential target for the treatment of cisplatin-resistant ovarian cancer. Mol Med Rep.

[22] Zhang Y, Ai H, Fan X, Chen S, Wang Y, Liu L (2020). Knockdown of long non-coding RNA HOTAIR reverses cisplatin resistance of ovarian cancer cells through inhibiting miR-138-5p-regulated EZH2 and SIRT1. Biol Res.

[23] Miao JT, Gao JH, Chen YQ, Chen H, Meng HY, Lou G: LncRNA ANRIL affects the sensitivity of ovarian cancer to cisplatin via regulation of let-7a/HMGA2 axis. Biosci Rep. 2019;39(7):BSR20182101. 10.1042/BSR20182101 PMC6609561 31189742

[24] Zhang D, Ding L, Li Y, Ren J, Shi G, Wang Y, Zhao S, Ni Y, Hou Y (2017). Midkine derived from cancer-associated fibroblasts promotes cisplatin-resistance via up-regulation of the expression of lncRNA ANRIL in tumour cells. Sci Rep.

[25] Chen L, Wang J, Liu Q (2022). Long noncoding RNAs as therapeutic targets to overcome chemoresistance in ovarian cancer. Front Cell Dev Biol.

[26] Lipsick JS (2010). The C-MYB story–is it definitive?. Proc Natl Acad Sci U S A.

[27] Ciciro Y, Sala A (2021). MYB oncoproteins: emerging players and potential therapeutic targets in human cancer. Oncogenesis.

[28] Srivastava SK, Khan MA, Anand S, Zubair H, Deshmukh SK, Patel GK, Singh S, Andrews J, Wang B, Carter JE (2022). MYB interacts with androgen receptor, sustains its ligand-independent activation and promotes castration resistance in prostate cancer. Br J Cancer.

[29] Salomoni P, Perrotti D, Martinez R, Franceschi C, Calabretta B (1997). Resistance to apoptosis in CTLL-2 cells constitutively expressing c-Myb is associated with induction of BCL-2 expression and Myb-dependent regulation of bcl-2 promoter activity. Proc Natl Acad Sci U S A.

[30] Zhang XY, Li YF, Ma H, Gao YH (2020). Regulation of MYB mediated cisplatin resistance of ovarian cancer cells involves miR-21-wnt signaling axis. Sci Rep.

[31] Tan WX, Sun G, Shangguan MY, Gui Z, Bao Y, Li YF, Jia ZH (2020). Novel role of lncRNA CHRF in cisplatin resistance of ovarian cancer is mediated by miR-10b induced EMT and STAT3 signaling. Sci Rep.

[32] Liu R, Zeng Y, Zhou CF, Wang Y, Li X, Liu ZQ, Chen XP, Zhang W, Zhou HH (2017). Long noncoding RNA expression signature to predict platinum-based chemotherapeutic sensitivity of ovarian cancer patients. Sci Rep.

[33] Xu J, Wu J, Fu C, Teng F, Liu S, Dai C, Shen R, Jia X (2018). Multidrug resistant lncRNA profile in chemotherapeutic sensitive and resistant ovarian cancer cells. J Cell Physiol.

[34] Vera O, Rodriguez-Antolin C, de Castro J, Karreth FA, Sellers TA (2018). Ibanez de Caceres I: An epigenomic approach to identifying differential overlapping and cis-acting lncRNAs in cisplatin-resistant cancer cells. Epigenetics.

[35] Li Q, Zhang J, Zhou J, Yang B, Liu P, Cao L, Jing L, Liu H (2018). lncRNAs are novel biomarkers for differentiating between cisplatin-resistant and cisplatin-sensitive ovarian cancer. Oncol Lett.

[36] Hussen BM, Azimi T, Hidayat HJ, Taheri M, Ghafouri-Fard S (2021). NF-KappaB interacting LncRNA: Review of its roles in neoplastic and non-neoplastic conditions. Biomed Pharmacother.

[37] Funato T, Satou J, Kozawa K, Fujimaki S, Miura T, Kaku M (2001). Use of c-myb antisense oligonucleotides to increase the sensitivity of human colon cancer cells to cisplatin. Oncol Rep.

[38] Tian M, Tian D, Qiao X, Li J, Zhang L (2019). Modulation of Myb-induced NF-kB -STAT3 signaling and resulting cisplatin resistance in ovarian cancer by dietary factors. J Cell Physiol.

[39] Xu QF, Tang YX, Wang X (2018). LncRNA EBIC promoted proliferation, metastasis and cisplatin resistance of ovarian cancer cells and predicted poor survival in ovarian cancer patients. Eur Rev Med Pharmacol Sci.

[40] Li Z, Niu H, Qin Q, Yang S, Wang Q, Yu C, Wei Z, Jin Z, Wang X, Yang A (2019). lncRNA UCA1 Mediates Resistance to Cisplatin by Regulating the miR-143/FOSL2-Signaling Pathway in Ovarian Cancer. Mol Ther Nucleic Acids.

[41] Wang DY, Li N, Cui YL (2020). Long non-coding RNA CCAT1 sponges miR-454 to promote chemoresistance of ovarian cancer cells to cisplatin by regulation of surviving. Cancer Res Treat.

[42] Xiao L, Shi XY, Li ZL, Li M, Zhang MM, Yan SJ, Wei ZL (2021). Downregulation of LINC01508 contributes to cisplatin resistance in ovarian cancer via the regulation of the Hippo-YAP pathway. J Gynecol Oncol.

[43] Wu J, Ni X, Yu Z, Wu S, Liu Z (2022). CRNDE inducing cisplatin resistance through SRSF1/TIA1 signaling pathway in ovarian cancer. Pathol Res Pract.

[44] Zhu Y, Yang L, Wang J, Li Y, Chen Y (2022). SP1-induced lncRNA MCF2L-AS1 promotes cisplatin resistance in ovarian cancer by regulating IGF2BP1/IGF2/MEK/ERK axis. J Gynecol Oncol.

[45] Yang T, Li S, Liu J, Yin D, Yang X, Tang Q (2018). lncRNA-NKILA/NF-kappaB feedback loop modulates laryngeal cancer cell proliferation, invasion, and radioresistance. Cancer Med.

[46] Huang X, Yan Y, Gui A, Zhu S, Qiu S, Chen F, Liu W, Zuo J, Yang L: A Regulatory Loop Involving miR-200c and NF-kappaB Modulates Mortalin Expression and Increases Cisplatin Sensitivity in an Ovarian Cancer Cell Line Model. Int J Mol Sci. 2022;23(23):15300. 10.3390/ijms232315300 PMC9737914 36499626

[47] Lv C, Ren C, Yu Y, Yin H, Huang C, Yang G, Hong Y: Wentilactone A Reverses the NF-kappaB/ECM1 Signaling-Induced Cisplatin Resistance through Inhibition of IKK/IkappaB in Ovarian Cancer Cells. Nutrients. 2022;14(18):3790. 10.3390/nu14183790 PMC9504226 36145166

[48] Knopfova L, Biglieri E, Volodko N, Masarik M, Hermanova M, Glaus Garzon JF, Ducka M, Kucirkova T, Soucek K, Smarda J (2018). Transcription factor c-Myb inhibits breast cancer lung metastasis by suppression of tumor cell seeding. Oncogene.

[49] Heuser C, Gattinoni L (2022). c-Myb redefines the hierarchy of stem-like T cells. Nat Immunol.

[50] Knopfova L, Benes P, Pekarcikova L, Hermanova M, Masarik M, Pernicova Z, Soucek K, Smarda J (2012). c-Myb regulates matrix metalloproteinases 1/9, and cathepsin D: implications for matrix-dependent breast cancer cell invasion and metastasis. Mol Cancer.

[51] Wang X, Wang Y, Gou S (2023). A platinum(II) complex HY1-Pt overcomes cisplatin-induced resistance and attenuates metastasis of epithelial ovarian cancer by cancer cell stemness inhibition. Int J Biochem Cell Biol.

[52] Qiu C, Ma Y, Wang J, Peng S, Huang Y (2010). Lin28-mediated post-transcriptional regulation of Oct4 expression in human embryonic stem cells. Nucleic Acids Res.

[53] Huang J, Lin H, Zhong M, Huang J, Sun S, Lin L, Chen Y (2018). Role of Lin28A/let-7a/c-Myc Pathway in Growth and Malignant Behavior of Papillary Thyroid Carcinoma. Med Sci Monit.

[54] Balzeau J, Menezes MR, Cao S, Hagan JP (2017). The LIN28/let-7 Pathway in Cancer. Front Genet.

