# Supplementary material for: Proto-oncogene c-Myb potentiates cisplatin resistance of ovarian cancer cells by downregulating lncRNA NKILA and modulating cancer stemness and LIN28A-let7 axis
Source: J Ovarian Res. 2024 May 14;17:102. doi: 10.1186/s13048-024-01429-w (PMC11092198; doi:10.1186/s13048-024-01429-w)
Supplement: Supplementary file 1 — Supplementary Material 1. [file 13048_2024_1429_MOESM1_ESM.docx]

**Supplementary Figure S1**

**
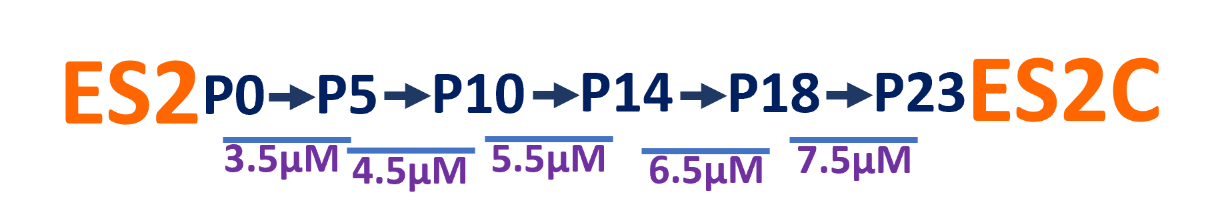
**

The cisplatin resistant ES2 cells (ES2C) were generated by long term exposure of the parental ES2 cells to cisplatin with gradual increase of cisplatin concentration. To start, ES2 cells were exposed to 3.5μM cisplatin (a sub-IC-50 dose) for five passages, followed by exposure to a slightly increased dose of 4.5μM cisplatin for next five passages, 5.5μM cisplatin for next four passages, 6.5μM cisplatin for next four passages and finally 7.5μM cisplatin for next five passages. The whole process took more than 4 months.

**Supplementary Table 1:** LncRNA levels in ES2C cells, relative to ES2 cells

| **LncRNA** | **Relative Levels** |
| --- | --- |
| ANRIL | 1.8±0.1 |
| BANCR | 2.2±0.1 |
| GAS5 | -1.9±0.2 |
| H19 | 2.2±0.2 |
| HOTAIR | 2.8±0.2 |
| LINC00982 | -2.0±0.1 |
| MALAT1 | 1.8±0.2 |
| MEG3 | -2.9±0.3 |
| NEAT1 | 3.1±0.3 |
| NKILA | -4.1±0.3 |
| SNHG3 | 2.5±0.1 |
| TUG1 | 2.1±0.2 |
| XIST | 1.7±0.1 |

The levels of lncRNAs were assessed by qRT-PCR in ES2 parental as well as ES2C (cisplatin resistant) cells. The levels in ES2 cells were rounded to ‘1’ and the relative levels in ES2C cells are presented in the Table. The presented results are representative of at least 3 different repeats.
